# Career adaptability of interpreting students: A case study of its development and interactions with interpreter competences in three Chinese universities

**DOI:** 10.3389/fpsyg.2022.974417

**Published:** 2022-09-14

**Authors:** Sha Tian, Zhining Zhang, Lingxiao Jia

**Affiliations:** ^1^School of Foreign Languages, Central South University, Changsha, China; ^2^School of Foreign Languages and Cultures, Ningxia University, Yinchuan, China

**Keywords:** career adaptability, interpreting students, interpreter competences, curriculum modification, MTI program

## Abstract

The issue of employability has already become a well-delineated topic of study among interpreting educators. However, the current literature still lacks descriptive research on interpreting students' employability development and ignores the developmental effects of interpreter competences in this process. Moreover, the advantage of using career adaptability for measurement is also under-researched. This exploratory case study aims at taking an initial step forward, surveying interpreting students' career adaptability development and the developmental effects of different interpreter competences on major adaptability resources, and ultimately diagnosing curriculum problems and making modifications accordingly. Thirty grade 2019 interpreting students from three Chinese universities contributed to data collection, through six questionnaires in a two-wave survey. The results highlight that, throughout the Chinese MTI program, interpreting students could become more concerned and well prepared for their future (concern), more curious to explore themselves and their surroundings (curiosity), and more capable of solving problems (confidence). The results also indicate that students' knowledge and language competence serve as the major facilitators in this process, and that other interpreter competences, such as psychological competence, transfer competence, professionalism, and cross-cultural competence, are also instrumental. In order to further boost their adaptability constructs, the results suggest that students' language and knowledge competence, professionalism, and cross-cultural and mental agility still need to be improved. Five suggestions for curriculum revision have been raised accordingly. As an initial effort, the current study will hopefully inspire further studies on interpreting students' career adaptability and add more knowledge to the curriculum design from this viewpoint.

## Introduction

Employability concerns an individual's ability to gain and maintain employment (Deng et al., 2021; Dinh et al., 2022). The issue of employability in Higher Education has become increasingly important in recent years, particularly at a time when the opportunities for employment growth are being jeopardized by the global COVID-19 outbreak (Boo et al., 2021). Specifically, interpreting students have grown concerned about their present and future career prospects given the pandemic's heavy toll on the professional environment (Ahrens et al., 2021; Gerber et al., 2021; Hoyte-West, 2022). Against such a backdrop, interpreting students need to build up more adaptability to unpredictable work in their careers. More attention should be paid to their employability, which can help them to cope with challenges and changes in the career construction process (Kundi et al., 2021; Maree, 2022). Given the importance of curriculum in education delivery (Campbell-Phillips, 2020), enhancing interpreting students' employability largely depends on the quality design of the interpreter training curriculum. In fact, as early as the 1990s, interpreting educators worldwide had already begun to develop relevant solutions. Since then, the relevant literature has increased substantially, and it has gradually become a well-delineated topic in interpreting studies.

Scholarly efforts have been presented from different perspectives. Some scholars attempted to assess the current curriculum of interpreter education, for example, adequacy of career preparation (Li, 2000, 2002, 2007; Gieure Sastre, 2016; Ma, 2017; Hu, 2018; Tang, 2020), integration of entrepreneurial learning (Klimkowski, 2015; Gieure Sastre, 2016; Rodríguez de Céspedes, 2017), the building of employability (Marzo-Navarro et al., 2009; Chouc and Calvo, 2010; Torres-Hostench, 2012; Cuminatto et al., 2017; Schnell and Rodríguez, 2017), or building of pre-professional identity (Salo et al., 2020). These studies generally confirmed the growing curriculum–industry gap and advocated for pedagogical modification. In response, other scholars delved into designing instructional activities underlying authentic experiential learning, for instance, simulations (Li, 2015; Kiraly and Massey, 2016; Van Egdom et al., 2020), team-based learning (Massey and Braendli, 2016), projects (Peverati, 2013; Santafé, 2014; Thelen, 2014), internships (Astley and Torres-Hostench, 2017; Li, 2018b), or mentorships (Olalla-Soler, 2018). Meanwhile, another important direction of scholarly research on this front has been concerned with market exploration, such as user expectation surveys (Pöchhacker, 1994; Diriker, 2011) or job ad analysis (Wang and Li, 2020).

Although there is a wealth of studies looking into this domain, important research gaps remain. First, focusing more on the curriculum–industry gap, the current literature still lacks holistic descriptive research on interpreting students' employability development under the current curriculum. This kind of research is significant, since it should be the basis of all related studies. Second, current studies ignore the competence-based nature of the interpreting training curriculum (Wang and Li, 2020; Oraki, 2022), paying little attention to the developmental effects of interpreter competences on students' employability. This research line is also crucial, as it could guide us to make important modifications based on the curriculum's core, rather than only adding external factors (such as entrepreneurial learning). In addition, some key concepts have rarely been applied in understanding the employability of interpreting students, for instance, career adaptability. As a central aspect of the career construction model of adaptation, this concept refers to the psychosocial construct that involves the individual's resources for dealing with current and anticipated career-related tasks, transitions, and traumas (Savickas and Porfeli, 2012; Boo et al., 2021). Being a crucial psychosocial resource for students, career adaptability functions as a vital indicator that could positively measure and predict their employability, such as future work engagement (Coetzee et al., 2017) and career outcomes (Kundi et al., 2021). Empirical analysis of this concept could not only offer an important data source to describe students' employability (Johnston, 2018; Klehe et al., 2021), but also serve as the logical starting point to identify possible enhancement solutions from a psychosocial perspective. Researchers worldwide have called for the use of career adaptability (Savickas and Porfeli, 2012; Zacher, 2014; Sverko and Babarovic, 2019; Klehe et al., 2021; Jia et al., 2022). As shown in a meta-analysis (Rudolph et al., 2017), it has already been frequently exploited to improve students' employability in various disciplines, such as business, sports, tourism, and hospitality, although still with a scarcity of empirical evidence in the interpreting literature. Thus, career adaptability, the guiding concept that may provide a theoretical and practical measurement framework, is also of great value for studying interpreting students' employability.

Future studies should work on the above-mentioned gaps. The current study hopes to take an initial step forward, aiming at describing interpreting students' employability development and its interactions with interpreter competence through the lens of career adaptability, and ultimately offering targeted suggestions for curriculum modification. To this end, set in the author's local educational context, this exploratory research intends (i) to measure interpreting students' career adaptability development throughout the program, (ii) to investigate the development effects of different interpreter competences on major career adaptability resources, and (iii) to identify problems in the existing curriculum and make modifications accordingly.

By expanding the use of career adaptability, the research results can offer implications for curriculum developers, instructors, and interpreting students. First of all, this study is of value to interpreting curriculum development and modification for curriculum developers and instructors. Next, the results may provide interpreting students with more knowledge about their career adaptability and its interactions with interpreter competence, thus helping them to undertake more focused preparations for their future career development.

## Theoretical framework

### Career adaptability

Career adaptability is a core aspect of the career construction model of adaptation. Derived from career construction theory, this model examines the individual's career construction process through links between the dimensions of adaptive readiness (psychological traits and personality), adaptability resources (career adaptability), adapting responses (performing adapting behaviors), and adaptation results (adaptive behavior outcomes) (Rudolph et al., 2017; Sverko and Babarovic, 2019). Career adaptability, as the adaptability resource in this model, refers to the psychosocial construct that assists individuals in coping with the unfamiliar, complex, and ill-defined problems presented by career tasks, occupational transitions, and work traumas (Savickas, 2005; Savickas and Porfeli, 2012; Akkermans et al., 2021). It is worth mentioning that these resources are not at the core of the individuals but reside at the intersection of person-in-environment, thus being defined as psychosocial (Savickas and Porfeli, 2012; Klehe et al., 2021).

As a set of psychosocial resources that condition adapting behaviors, career adaptability is a hierarchical construct that comprises a four-dimensional framework of adaptability: concern, control, curiosity, and confidence (Savickas, 2005; Savickas and Porfeli, 2012; Myszkowski et al., 2022). Concern consists of the ability to be aware of and prepare for future career tasks. Control reflects the belief in one's personal responsibility for one's career development, as well as the perceived personal control over constructing one's career. Curiosity reflects the tendency to explore one's possible selves and future scenarios so as to facilitate a good fit between oneself and the future environment. Confidence is the self-confidence in developing one's ability to solve career-related problems and pursue personal aspirations (Savickas and Porfeli, 2012; Rudolph et al., 2017; Sverko and Babarovic, 2019; Myszkowski et al., 2022). These psychosocial resources are proven to be crucial self-regulatory elements in directing future-oriented actions and proactive career behaviors (Taber and Blankemeyer, 2015; Klehe et al., 2021).

In the context of the career construction model of adaptation, career adaptability acts as a mediator in the relationship between individuals' adaptive readiness and adaptive response, shaping adaptive strategies aimed at achieving adaptation results (Hirschi and Valero, 2015; Sverko and Babarovic, 2019; Korkmaz, 2022). When people are confronted with complicated or difficult tasks related to career development, they tend to draw from their ability to adapt in order to solve problems and take change-oriented actions to adapt to the changing environment (Savickas and Porfeli, 2012; Korkmaz, 2022). Given its engagement in the career construction process, the wider career literature has highlighted the central role played by career adaptability in individuals' employability, conceptualized as enhancing proactive career behaviors and increasing the possibility of employment (Taber and Blankemeyer, 2015; Coetzee et al., 2017; Klehe et al., 2021; Kundi et al., 2021). Thus, it is of great value to study interpreting students' employability from this angle.

### Interpreter competences

Interpreter employment requires graduate students to be equipped with professional interpreter competences (Astley and Torres-Hostench, 2017; Crezee and Marianacci, 2022). Interpreting curriculum developers seek to provide adequate coverage of an agreed set of interpreter competences in most interpreting training programs (Wang and Li, 2020; Oraki, 2022). The core of the interpreter training curriculum is to help students to acquire these interpreter competences (Schnell and Rodríguez, 2017). Thus, when measuring interpreting students' career adaptability under the current curriculum, one indispensable aspect is to explore the developmental effects of different interpreter competences on the major adaptability resources. For the purpose of this study, it is also necessary to devise an interpreter competences framework based on the relevant literature.

According to Kiraly (2000), interpreter competences refer to the framework of declarative knowledge, procedural knowledge, and attitudes essential to accomplish a professional interpreting task. Based on the relevant literature, interpreter competences can be divided into seven categories, which could again be broken down into 23 component skills that apply to both consecutive and simultaneous interpreting. Details of the interpreter competences' components can be illustrated as follows: **language competence** (a. analytical listening and comprehension, and b. linguistic agility) (Kiraly, 2000; Pöchhacker, 2004; Gile, 2009; Albl-Mikasa, 2013; Li, 2018b; Bezzaoucha, 2021), **transfer competence** (c. concentration, d. working memory, e. linguistic transfer, f. multi-tasking between listening, analysis, note-taking, production, and monitoring, g. monitoring of target language production quality, and h. elegant delivery of message in the target language) (Kiraly, 2000; Kalina, 2002; Pöchhacker, 2004; Gile, 2009; Li, 2018b; Bezzaoucha, 2021), **knowledge competence** (i. general know-how and j. subject-specific knowledge) (Kiraly, 2000; Pöchhacker, 2004; Gile, 2009; Schnell and Rodríguez, 2017; Li, 2018b; Bezzaoucha, 2021), **cross-cultural competence** (k. knowledge about different cultures, l. awareness of bridging the cross-cultural differences, and m. sensitivity to the communicative context) (Kiraly, 2000; Pöchhacker, 2004; Gile, 2009; Kuznik and Hurtado Albir, 2015; Li, 2018b; Bezzaoucha, 2021), **strategic competence** (n. automatic use of language-specific strategies, o. culture-specific strategies, and p. on-site problem-oriented strategies) (Kiraly, 2000; Pöchhacker, 2004; Gile, 2009; Li, 2018b; Bezzaoucha, 2021), **psychological competence** (q. being calm under pressure and r. mental agility) (Kiraly, 2000; Pöchhacker, 2004; Gile, 2009; Li, 2018b), and **professionalism** (s. preparation, t. professional ethics, u. interpersonal relations and team work, v. meta-reflection, and w. life-long learning) (Kiraly, 2000; Pöchhacker, 2004; Gile, 2009; Feinauer and Lesch, 2013; Li, 2018b; Bezzaoucha, 2021).

## Research questions and research design

Three sets of questions drove the current research: (1) How does interpreting students' career adaptability develop throughout the MTI program? (2) What are the developmental effects of different interpreter competences on major career adaptability resources throughout the MTI program? (3) What are the problems in the existing competence-based curriculum in terms of career adaptability development? What suggestions could be raised accordingly? By answering the above questions, the current study aims at demonstrating what needs to be taken into account for career adaptability development when designing and renewing the interpreting curriculum.

As for the research design of question one, it employed a two-wave questionnaire survey approach, aiming to compare students' career adaptability at the beginning and end of the interpreter training program. The reason why the researchers chose the pre-/post-program design was based on its extensive application in the measurement of change. According to the wider literature, pre–post design is widely used in behavioral research in various fields for the purpose of measuring change resulting from experimental treatments, including the field of education (Dimitrov and Rumrill, 2003; Shek and Sun, 2012; Clifford et al., 2021). Thus, the current study tried to collect the pre/post-program data for analyzing interpreting students' development of career adaptability. Specifically, prior to the program, participants completed the first-wave questionnaire (Career Adapt-Abilities Scale), providing quantitative data on their baseline career adaptability. At the end of the program, the same questionnaire was administered again to the participants, aiming to collect data on their developed career adaptability. A descriptive and comparative analysis of the pre-/post-program data generated students' career adaptability gains throughout the program.

For research questions two and three, it used a self-assessment questionnaire survey at the end of the program. These questionnaires were designed by the researchers based on the framework of career adaptability and interpreter competences. Specifically, after students finished the questionnaire for research question one in the second stage, they were asked to complete several questionnaires to assess by themselves which interpreter competence components were important (and to what degree) to the four adaptability resources, and which ones they were still inadequate in and hence impeded their development. A descriptive and inferential analysis of the dataset helped to visualize the developmental effects and the corresponding problems.

The reasons that the researchers designed a self-assessment survey in the second stage were 2 fold. First, it is difficult to measure the developmental effects on career adaptability in a standardized way (for instance, a test), particularly for separate measurement. Second, as proven by many studies (Combs et al., 2008; Lee, 2011; Fernández and Zabalbeascoa, 2012; Brown et al., 2014; Li, 2018a; Winke et al., 2022), correlations between self-assessments and objective measures are of the same degree as those between different subsections in a standardized direct test.

## Methodology

### Context

**Figure 1:**
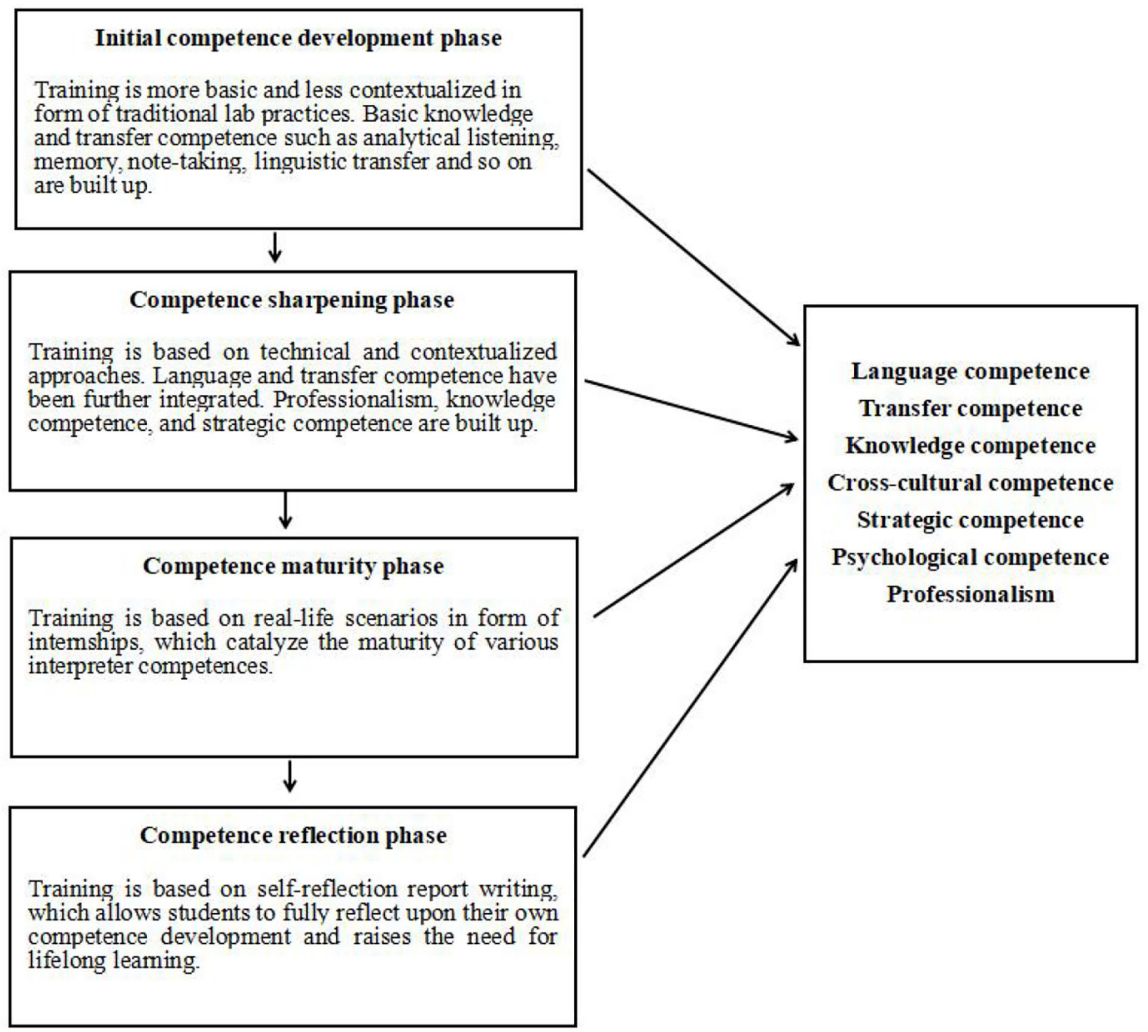
Four phases of the competence-based interpreting curriculum in China.

The present research was undertaken in three Chinese universities offering MTI programs (Master of Translation and Interpreting), with a special focus on the interpreting track. Chinese MTI programs are operated under the unified guidance of the National Advisory Committee for MTI Training convened by the Ministry of Education. According to its 2011 Revised Edition of Recommended Program Plan, the interpreting track aims at training students to become competent interpreters who are able to offer high-level consecutive and simultaneous interpreting services between English and Chinese. Hence, concerning the curricula provided by different Chinese universities, although slightly different in some courses or module settings, they are basically under the same format, underpinned by the training of interpreter competences. The three participating universities were no exception. As illustrated in Figure 1, the competence-based interpreting curriculum under Chinese MTI programs can be divided into four phases: initial competence development in traditional lab practices, competence sharpening through simulated and advanced practices, competence maturity *via* internships, and competence reflection in the form of thesis writing (Cai et al., 2015; Li, 2015; Tang, 2020; Wu and Jiang, 2021).

In the first two phases, students mainly take a series of interpreting courses. It usually takes them 1 year or so to complete these courses. Specifically, courses in the first phase, or the initial competence development phase, are more basic and less contextualized (Li, 2015; Tang, 2020). For instance, courses such as sight translation and interpreting basics are offered to build up basic interpreting knowledge and skills, such as analytical listening, memory, note-taking, linguistic transfer, and so on. Courses in the second phase, or competence sharpening phase, evolve from general to more technical and contextualized approaches. To illustrate, courses such as consecutive interpreting and simultaneous interpreting are taught to further integrate and sharpen the skills built in the basic courses; courses such as mock conferences are organized to offer simulated projects for the development of professionalism, strategic competence, psychological competence etc.; courses such as business interpreting and diplomatic interpreting help students in interpreting in a specific domain, endowing them with essential subject knowledge through contextualized learning (Hoffman and Kiraly, 2014; Li, 2015; Wu and Jiang, 2021). However, there are no language enhancement courses, because students are believed to have adequate linguistic competence to begin interpreting training.

The third phase, or competence maturity phase, takes place outside of classrooms, through internships. Students need to undertake interpreting internships for at least one semester. When they begin to show the ability to cope with authentic tasks after the first year of training, internships pave the way to professional realism and real-life scenarios, which improve the maturity of their interpreter competences (Li, 2015; Tang, 2020; Wu and Jiang, 2021). Then, the fourth phase, or competence reflection phase, occurs within students' thesis writing. Students are required to write a practice report based on their own interpreting project accomplished during the internships. This allows them to fully reflect upon their own competence development and highlight the need for future planning and lifelong learning (Fernández Prieto and Sempere Linares, 2010; Tang, 2020).

### Participants

Participants of the current study were chosen by a purposive sampling method. Thirty grade 2019 interpreting students from the three chosen Chinese universities participated in the current study. Table 1 shows the descriptive profiles of the three chosen institutions for this study. They are located in different parts of China. All are listed in the “Double First-Class” university project. All have a relatively long history in operating MTI programs in China. Moreover, given the fact that MTI programs in China are operated within similar competence-based curriculum frameworks, as shown above, students from the three major universities, namely, Ningxia University in northwest China, Central South University in central China, and Hainan University in the south of China, are somewhat diverse to represent the general landscape. Although the sample was small, it was still representative to some degree.

**Table 1:** Descriptive profiles of the three participating Chinese universities.

**Participating Universities**	**Description**
Ningxia university	located in Yinchuan, Ningxia province, northwest of China, listed in National “Project 211” and “Double First-Class” university project, authorized to operate MTI programs since 2010.
Central South university	located in Changsha, Hunan province, central of China, listed in National “Project 985” and “Double First-Class” university project, authorized to operate MTI programs since 2007.
Hainan university	located in Haikou, Hainan province, south of China, listed in National “Project 211” and “Double First-Class” university project, authorized to operate MTI programs since 2010.

The profile of participants from the three chosen universities in terms of the quantitative component is presented in Table 2. They were grade 2019 interpreting students. When this research project was initiated, all had acquired a certain level of proficiency in English by completing their undergraduate studies and were ready to begin receiving interpreter training under the MTI programs offered by their home universities. With regard to gender, there were 7 (23.3%) male students and 23 (76.7%) female students. They were all in their early twenties, with 5 (16.7%) aged 21 years old, 23 (76.7%) aged 22 years old, and 2 (6.6%) aged 23 years old. Ten (33.3%) came from Ningxia University, 11 (36.7%) from Central South University, and 9 (30%) from Hainan University. All were informed of the purpose of the study and participated in the study voluntarily and anonymously. Ethical approval was also obtained from the ethics committees of the participating universities.

**Table 2:** Profile of the participants (quantitative).

	**Gender**						
		**Male**			**Female**		
		**Age category**			**Age category**		
		21	22	23	21	22	23
**University**	Ningxia university	0	1	1	1	7	0
	Central South university	1	2	0	1	7	0
	Hainan university	1	1	0	1	5	1

### Instruments

Six questionnaires were used to address the research questions (https://doi.org/10.6084/m9.figshare.20443563), one on students' career adaptability development, four on developmental effects of different interpreter competences on major adaptability resources, and one on the problems and modifications (Table 3).

**Table 3:** Instruments.

**Research questions**	**Instruments**
**Research question one**: interpreting students' career adaptability development throughout the program.	Career Adapt-Abilities Scale
**Research question two**: developmental effects of different interpreter competences on major adaptability resources throughout the program.	A set of four questionnaires designed to select and rate the importance of each interpreter competence component toward the development of four adaptability resources, respectively.
**Research question three**: problems and suggestions for the existing competence-based curriculum.	A mixed questionnaire designed to collect both quantitative data on students' competence inadequacy (which hence impeded their career adaptability development) and qualitative data on their corresponding suggestions.

Career adaptability could be assessed by diverse scales that contain various adaptability components, such as the Career Development Inventory (CDI; Super et al., 1988), the Adult Career Concerns Inventory (ACCI; Super et al., 1988), and the Career Adapt-Abilities Scale (CAAS; Savickas and Porfeli, 2012; Maggiori et al., 2017). Among them, the CAAS has proven to be the most extensively used operationalization of the construct (Kirchknopf, 2020; Sou et al., 2021). It has been widely used with diverse groups of university students and demonstrated measurement equivalence in cross-national research (Savickas and Porfeli, 2012; Johnston et al., 2013; Oncel, 2014; Sou et al., 2021; Marques et al., 2022). Hence, to address the first research question, this study applied the CAAS for assessment. The CAAS consists of 24 items in four dimensions that measure the psychosocial adaptability resources of concern, control, curiosity, and confidence, with six items each. All items are rated employing a scale from 1 (not strong) to 5 (strongest). The total score yielded indicates a person's career adaptability. Savickas and Porfeli (2012) reported coefficients of 0.83, 0.74, 0.79, and 0.85 for the sub-scales of concern, control, curiosity, and confidence, respectively. For this sample, Cronbach's alpha was 0.83 for concern, 0.84 for control, 0.88 for curiosity, and 0.89 for confidence.

A set of four questionnaires was designed to collect data on the second research question. They were designed based on the framework of career adaptability and interpreter competences. They were in the same format, consisting of two parts. In the first part, interpreting students were asked to select, from a list of 23 interpreter competence components under seven categories, as illustrated in Table 4, the ones that they deemed important to the development of adaptability resources of concern (questionnaire one), control (questionnaire two), curiosity (questionnaire three), and confidence (questionnaire four). Based on their choices, in the second part, they were required to rate the importance of each component toward the development of concern (questionnaire one), control (questionnaire two), curiosity (questionnaire three), and confidence (questionnaire four) on a 5-point Likert scale: “1 Very unimportant,” “2 Unimportant,” “3 Neutral,” “4 Important,” and “5 Very important.” Five participants and three colleagues were asked to comment on its validity and comprehensibility during piloting. A corresponding revision was made.

**Table 4:** List of interpreter competences components.

**Interpreter competences**	**Component skills**
Language competence	a. Analytical listening and comprehension b. Linguistic agility
Transfer competence	c. Concentration d. Working memory e. Linguistic transfer f. Multi-tasking between listening, analysis, note-taking, production and monitoring g. Monitoring of target language production quality h. Elegant delivery of message in the target language
Knowledge competence	i. General know-how j. Subject-specific knowledge
Cross-cultural competence	k. Knowledge about different cultures l. Awareness of bridging the cross-cultural differences m. Sensitivity to the communicative context
Strategic competence	n. Automatic use of language specific strategies o. Culture-specific strategies p. On-site problem-oriented strategies
Psychological competence	q. Being calm under pressure r. Mental agility
Professionalism	s. Preparation (glossary building, background information research, etc.) t. Professional ethics (dressing code, behavior, sense of responsibility etc.) u. Interpersonal relations, team work, etc. v. Meta-reflection (strengths, weaknesses, challenges, etc.) w. Life-long learning

Another mixed questionnaire was designed to answer the third research question. It consisted of two parts. In the first part, interpreting students were asked to select, from the same list, the competences components that they still felt inadequate in and hence impeded the development of their career adaptability. The second part was presented as an open-ended question used to validate some results of the study: “In view of further improving your career adaptability, what suggestions do you have for the curriculum modification?” A small group pilot was also conducted before the formal survey. A revision was made accordingly.

### Procedure of the data collection

A longitudinal procedure was followed to collect the data. As seen in the research design, it generally consisted of two phases.

In the first phase, the CAAS together with the consent statement was delivered by the researchers to all grade 2019 interpreting students of the three Chinese universities in early September 2019, during the first week of the MTI programs at their home universities. Specifically, on 3 September 2019, the researchers visited Ningxia University. After gaining approval from its ethics committee, the researchers gathered the participants in one classroom and distributed the paper CAAS to them. Before completing the questionnaires, the researchers discussed all 24 items with the students to ensure that they understood them well, so as to improve the accuracy. After the survey, the researchers collected all the paper questionnaires uniformly. The whole process took 20 min. Similar investigation proceedings also took place at Central South University on 6 September 2019 and at Hainan University on 10 September 2019. Then, all participating students received interpreter training at their home institutions under a similar competence-based curriculum. Throughout their MTI programs, they undertook various interpreting-related courses, participated in internships, and wrote their theses.

When the participants were ready to graduate, the second phase began. In June 2021, the researchers visited Ningxia University again and gathered the participants in one classroom. At this stage, they were asked to complete three waves of questionnaires for the study. For the first wave, the paper CAAS was administered again to obtain data on their developed career adaptability. Similarly, the researchers discussed all items with the students before their completion. After the survey, the researchers collected all the paper CAAS forms and the whole process took 20 min. For the second wave, a set of four questionnaires were given to them to gain data on the developmental effects of different interpreter competences regarding the four adaptability resources one by one. To maximize the response accuracy, the researchers clarified the definition of each resource through verbal interpretation each time before the students made their own choices. After the survey, the researchers collected all the paper questionnaires, and the whole process took around 40 min. For the third wave, the mixed questionnaire was applied to collect both quantitative and qualitative data about the existing problems and suggestions. Similarly, the researchers elaborated for the students all the necessary information, for instance, the definition of career adaptability, the survey intention, etc., before they filled in the questionnaires. After the survey, the researchers collected all the questionnaires, and the whole process took around 20 min. Altogether, it took around 80 min to complete all the investigations in the second stage. Similar survey proceedings also took place at Central South University and Hainan University during their graduation season.

### Data analysis

All data collected were statistically analyzed in line with the research questions.

For research question one, comparing interpreting students' CAAS scores at the beginning and end of training could indicate their career adaptability development throughout the MTI program. SPSS 22 was used to analyze the data. A descriptive and comparative analysis was applied from the results of the CAAS prior to and after the program, indicated on the 5-point Likert scale and through the outcomes from the *T*-test.

For research question two (exploring the developmental effects of interpreter competences on career adaptability), statistical analysis was performed on two fronts. Data from the first part of the four questionnaires were counted by the author manually. Simple frequencies and percentages (selection rate) were calculated. SPSS 22 was used to perform a descriptive inferential analysis for data from the second part of the four questionnaires. The mean importance of each component was calculated.

For research question three, statistical and content analyses were performed. To diagnose the problems in the current competence-based curriculum, the author counted the data from the first part of the mixed questionnaire manually. Simple frequencies and percentages (selection rate) were calculated. To imply corresponding suggestions, the mean selection rate of the importance of and the inadequacy in each component was entered into SPSS 22, together with letters representing different components, to draw a scatterplot graph. Furthermore, a thematic analysis was conducted in categorizing students' qualitative responses to the open-ended question of the mixed questionnaire. To safeguard the accuracy, this process was checked by two colleagues with qualitative research experiences.

All the above findings were then analyzed and discussed to make specific implications for curriculum modification.

## Results and discussion

### Career adaptability development

**Figure 2:**
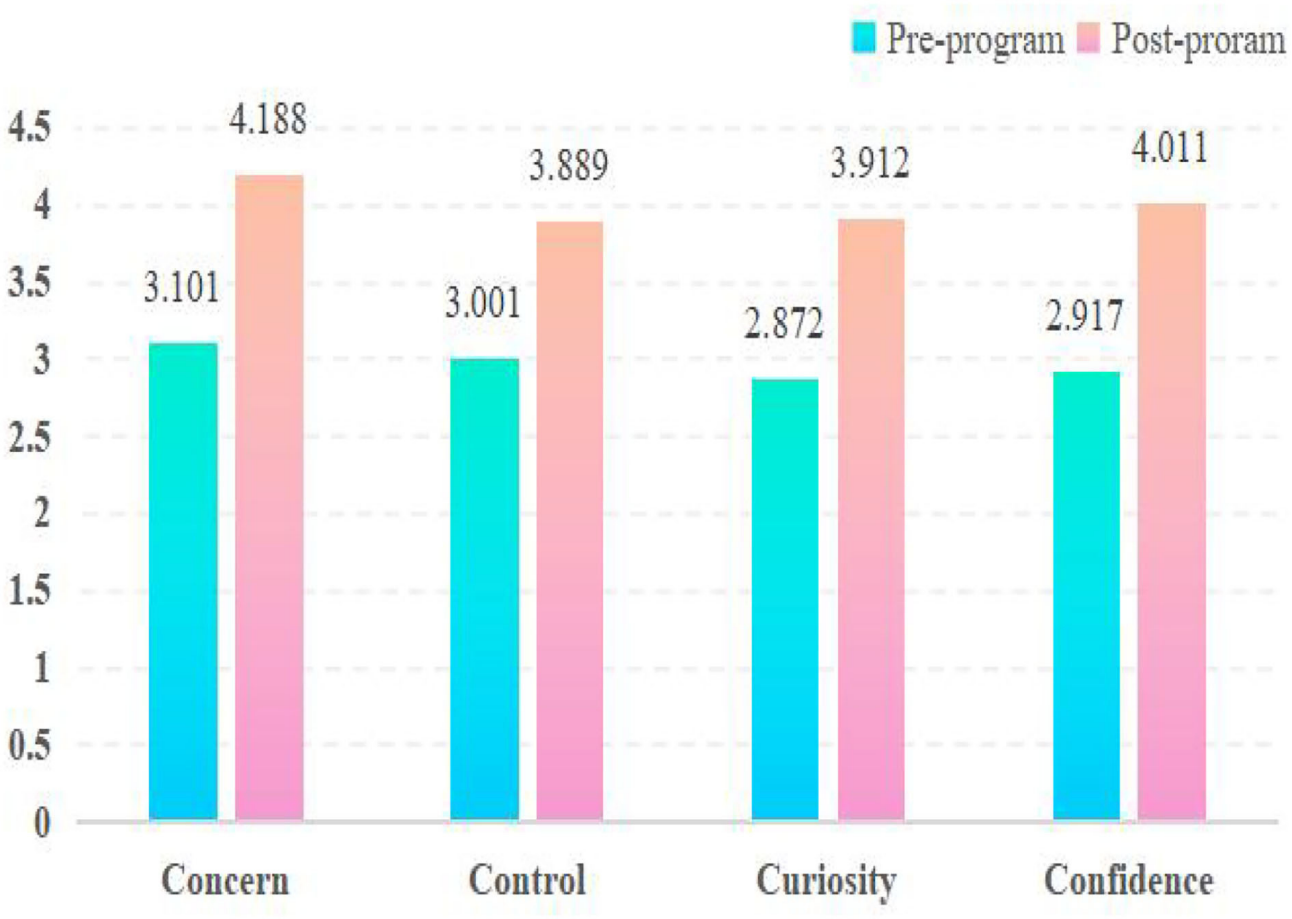
Pre-/post-program mean values for four CAAS second-order adaptability resources.

In order to measure interpreting students' career adaptability development, the CAAS was administered twice, as elaborated above. Before and after attending the MTI programs, participants rated each of the 24 items in four psychosocial dimensions on a scale ranging from 1, not strong, to 5, strongest. All the analyses were conducted using SPSS 22. The pre-/post-program mean values for each first-order indicator were calculated, based on which students' gains in each item were also measured (Table 5). The pre/post-program mean values for the four second-order indicators were also estimated (Figure 2).

**Table 5:** Pre/post-program mean values for CAAS first-order indicators.

**Adaptability resources**		**Pre-program means**	**Post-program** **means**	**Paired samples *t*-test**	**Pre- post-program gains**
Concern	1. Thinking about what my future will be like	3.133	4.200	0.001	1.067
	2. Realizing that today's choices shape my future	3.733	4.767	0.001	1.034
	3. Preparing for the future	2.933	3.867	0.001	0.934
	4. Becoming aware of the career choices	2.733	3.861	0.001	1.128
	5. Planning how to achieve my goals	2.767	3.900	0.001	1.133
	6. Concerned about my career	3.367	4.533	0.001	1.166
Control	1. Keeping upbeat	3.133	3.633	0.001	0.5
	2. Making decisions by myself	3.067	4.200	0.001	1.133
	3. Taking responsibility for my action	3.100	4.233	0.001	1.133
	4. Sticking up for my beliefs	2.967	3.633	0.001	0.666
	5. Counting on myself	3.000	4.067	0.001	1.067
	6. Doing what's right for me	2.767	3.567	0.001	0.8
Curiosity	1. Exploring my surroundings	2.600	3.533	0.001	0.933
	2. Looking for opportunities to grow as a person	3.267	4.300	0.001	1.033
	3. Investigating options before making a choice	2.833	4.000	0.001	1.167
	4. Observing different ways of doing things	2.667	3.567	0.001	0.9
	5. Probing deeply into questions I have	2.633	3.800	0.001	1.167
	6. Becoming curious about new opportunities	3.233	4.367	0.001	1.134
Confidence	1. Performing tasks efficiently	2.700	3.967	0.001	1.267
	2. Taking care to do things well	3.000	4.133	0.001	1.133
	3. Learning new skills	2.933	4.100	0.001	1.167
	4. Working up to my ability	3.033	4.300	0.001	1.267
	5. Overcoming obstacles	2.733	3.800	0.001	1.067
	6. Solving problems	2.700	3.767	0.001	1.067

As shown in Table 5 and Figure 2, interpreting students had made different degrees of progress in all adaptability items throughout the programs, suggesting that they did obtain development in their career adaptability within the current interpreting curriculum framework. A detailed examination presented some interesting findings.

Interpreting students' pre-program concerns had already been strong (M = 3.101) (Figure 2), particularly Conc1 (M = 3.133), Conc 2 (M = 3.733), and Conc 6 (M = 3.367) (Table 5), implying that they were concerned about their career futures but did not know how to plan successfully before attending the program. Significant progress had been made in all concern items after training, particularly in Conc 6 and Conc 5 (Table 5), and their concern had evolved to be very strong (M = 4.188, the highest among the four scales) (Figure 2). The results suggested that students had become increasingly aware of their vocational futures and learned to plan and prepare to achieve their goals throughout the programs.

Interpreting students' pre-program control was strong (M = 3.001) (Figure 2) as well, particularly in Cont 1(M = 3.133) and Cont 3 (M = 3.100) (Table 5), showcasing that they were optimistic about their vocational futures and willing to bear the relevant responsibilities before attending the program. Although progress was also made in all control items during training, Cont 1 (Gain = 0.5), Cont 4 (Gain = 0.666), and Cont 6 (Gain = 0.8) were the ones in which the least gains had been obtained (Table 5). In particular, the contrasting results of Cont 1, which was comparatively higher before but rose the least afterward, demonstrated that students were not as positive about their careers as they were expected to be after the program. This was also triangulated by the post-program mean of control, 3.889 (Figure 2), which remained strong but not very strong. One possible explanation might be that the global COVID-19 outbreak and other increasing uncertainties have somewhat dampened students' beliefs and perceived personal control over their future careers.

Interpreting students' pre-program curiosity was not particularly strong (M = 2.872) (Figure 2), particularly in Curi 1 (M = 2.600), Curi 4 (M = 2.667), and Curi 5 (M = 2.633) (Table 5), illustrating that they were not, especially, conscious of exploring their possible selves and future scenarios before attending the program. Significant progress had been made in all curiosity items after training, particularly in Curi 3 (Gain = 1.167), Curi 5 (Gain = 1.167), and Curi 6 (Gain = 1.134), which were among the six items with the greatest gains (Table 5). As a result, students' post-program mean of curiosity was upgraded to 3.912 (Figure 2), nearly becoming very strong. The results revealed that students gradually achieved a good fit between themselves and the future environment throughout the program.

Students' pre-program curiosity was not especially strong (M = 2.917) either (Figure 2), particularly in Conf 1 (M = 2.700), Conf 5 (M = 2.733), and Conf 6 (M = 2.700), indicating that they were unable to solve their career-related problems before attending the program. Significant progress had been made in all confidence items after training. In particular, Conf 1 (Gain = 1.267), Conf 3 (Gain = 1.167), and Conf 4 (Gain = 1.267) were the areas where most progress had been made (Table 5). These inspiring data illustrate that students developed the abilities needed to tackle concrete career problems and built confidence on this front *via* the program. Consequently, their post-program mean of confidence reached 4.011 (Figure 2), considered very strong, second only to concern.

To summarize, throughout the MTI programs, interpreting students became more concerned about and well prepared for their future careers (concern), displayed growing curiosity to explore themselves and their surroundings (curiosity), and built up their abilities to face and solve problems (confidence). Nevertheless, students' perceived optimism, belief, and control over constructing their careers (control) failed to develop to a satisfactory level.

### Developmental effects of interpreter competences on career adaptability

A set of four questionnaires were designed to collect data on this question. At the end of the MTI programs, participants were first asked to select from a list of 23 competence components (Table 4) that had facilitated their development of concern (questionnaire one), control (questionnaire two), curiosity (questionnaire three), and confidence (questionnaire four), and then rate the importance of each component on a 5-point Likert scale. Simple selection rates were calculated by the authors manually, and SPSS 22 was used to perform a descriptive analysis of the mean importance of each component. Details are illustrated in Tables 6–**9**. It is worth mentioning that, given the statistical fact that the mean selection rate of the whole dataset was calculated as around 59.06%, only those components selected by at least 59.06% are listed and considered to be comparatively significant.

**Table 6:** Frequently selected components that contributed to the development of concern.

**Interpreter competences components**	**Selection rate (%)**	**Mean of importance**
v. Meta-reflection	90	4.333
s. Preparation	86.67	4.267
i. General know-how	83.33	4.200
b. Linguistic agility in English and Chinese	80	4.100
t. Professional ethics	76.67	4.067
r. Mental agility	76.67	4.033
u. Interpersonal relations, team work, etc.	73.33	4.000
a. Analytical listening and comprehension	70	3.933
w. Life-long learning	66.67	3.867
c. Concentration	66.67	3.833
j. Subject-specific knowledge	60	3.667

As presented in Table 6, the participants mainly selected 11 components that had significantly improved their development of concern. They, respectively, belong to four categories of competences, namely, professionalism, knowledge competence, language competence, and psychological competence. Components v, s, i, b, t, r, and u had a mean of importance above four (important) and were selected by more than 73.33% of the students. Among the top components, most were elements of professionalism (components v, s, t, and u), highlighting the instrumental role of professionalism in advancing students' concerns and planning for their future careers. Moreover, knowledge, language, and psychological competence also play a part in this process.

As displayed in Table 7, the participants mainly selected 15 components that had greatly improved their development of control. The ones that they felt most important were components i, b, e, j, and a (with a mean of importance above 4 and selected by more than 76.67%), the core skills of knowledge competence, language competence, and transfer competence. Most others were also related to transfer competence (as signified by components h, c, r, d, f, and g), which were rather fundamental in interpreter training. These findings provide evidence that adequate linguistic and interpreting skills plus encyclopedia knowledge could enhance students' perceived beliefs and personal control regarding their vocational futures. Hence, to address the unsatisfactory development of control, greater language and knowledge enhancement for students is needed throughout the programs.

**Table 7:** Frequently selected components that contributed to the development of control.

**Interpreter competences components**	**Selection rate (%)**	**Mean of importance**
i. General know-how	93.33	4.400
b. Linguistic agility in English and Chinese	90	4.333
e. Mastering of necessary transfer skills	83.33	4.167
j. Subject-specific knowledge	83.33	4.133
a. Analytical listening and comprehension	76.67	4.033
h. Elegant delivery in the target language	76.67	3.933
q. Being calm under pressure	73.33	3.900
c. Concentration	73.33	3.867
v. Meta-reflection	73.33	3.833
t. Professional ethics	70	3.733
r. Mental agility	66.67	3.667
d. Working memory	66.67	3.633
f. Multi-tasking	66.67	3.600
g. Monitoring of target language production	66.67	3.533
n. Automatic use of language strategies	60	3.467

As shown in Table 8, participants mainly selected 16 components that had substantially improved their development of curiosity. They nearly covered all seven categories of interpreter competence. Among them, the top six components (components i, k, j, m, b, and r) had a mean of importance above four and were selected by more than 76.67% of students, demonstrating the great instrumental value of knowledge competence, cross-cultural competence, and language competence in this regard. The findings show that these competence can spark students' desire to explore their possible selves and their future scenarios. This is generally consistent with the literature that upholds the value of cross-cultural awareness training, language enhancement, and knowledge enrichment in MTI programs (Li, 2016; Benjamin et al., 2021; Reichertz, 2021).

**Table 8:** Frequently selected components that contributed to the development of curiosity.

**Interpreter competences components**	**Selection rate (%)**	**Mean of importance**
i. General know-how	93.33	4.433
k. Knowledge about different cultures	86.67	4.300
j. Subject-specific knowledge	80	4.167
m. Sensitivity to the communicative context	76.67	4.067
b. Linguistic agility in English and Chinese	73.33	4.033
r. Mental agility	73.33	4.000
l. Awareness of bridging the cross-cultural differences	70	3.900
a. Analytical listening and comprehension	66.67	3.867
e. Mastering of necessary transfer skills	66.67	3.833
o. Culture-specific strategies	66.67	3.800
w. Life-long learning	66.67	3.767
p. On-site problem-oriented strategies	63.33	3.733
v. Meta-reflection	63.33	3.700
c. Concentration	60	3.600
q. Being calm under pressure	60	3.567
u. Interpersonal relations, team work, etc.	60	3.500

As manifested in Table 9, participants mainly selected 21 components that had considerably improved their development of confidence. They nearly covered all the components under the seven categories of interpreter competences, which means that all have played a crucial and coordinated role in building students' ability and confidence to face and solve career-related problems. Among them, special attention could be paid to components b, e, a, i, v, k, and p, the mean importance of which was above four. It is also interesting to note that the most influential competence is language competence, as evidenced by components b (with the selection rate of 93.33% and the mean importance of 4.433) and a (with the selection rate of 86.67% and the mean importance of 4.333), which ranked among the top three. This finding reveals that language competence serves as the main contributor to students' confidence and should be paid more attention to in MTI programs.

**Table 9:** Frequently selected components which contributed to development of confidence.

**Interpreter competences components**	**Selection rate (%)**	**Mean of importance**
b. Linguistic agility in English and Chinese	93.33	4.433
e. Mastering of necessary transfer skills	90	4.400
a. Analytical listening and comprehension	86.67	4.333
i. General know-how	86.67	4.300
w. Life-long learning	83.33	4.233
v. Meta-reflection	76.67	4.067
k. Knowledge about different cultures	76.67	4.033
p. On-site problem-oriented strategies	73.33	4.000
r. Mental agility	73.33	3.933
t. Professional ethics	73.33	3.867
n. Automatic use of language strategies	70	3.900
f. Multi-tasking	70	3.833
o. Culture-specific strategies	70	3.767
h. Elegant delivery in the target language	66.67	3.767
d. Working memory	66.67	3.733
c. Concentration	66.67	3.667
j. Subject-specific knowledge	66.67	3.667
m. Sensitivity to the communicative context	66.67	3.667
q. Being calm under pressure	63.33	3.633
g. Monitoring of target language production	60	3.600
u. Interpersonal relations, team work, etc.	60	3.567

In addition, to integrate the above four datasets and calculate the mean selection rate and importance of each, a comprehensive view of the developmental effects of different interpreter competences on career adaptability could be obtained. As presented in Table 10, 16 components whose mean selection rates were over 60% are significantly conducive to students' career adaptability. They nearly cover all seven categories. Given the top four ranking of components i (with the mean selection rate of 85.53% and mean importance of 4.333), b (with the mean selection rate of 83.33% and mean importance of 4.223), a (with the mean selection rate of 76.67% and mean importance of 4.140), and j (with the mean selection rate of 74.17% and mean importance of 4.013), knowledge and language competence are of overriding value in this process. Moreover, students' psychological competence (mainly components r and q), transfer competence (mainly components c, e, and d), professionalism (mainly components u, v, t, s, and w), and cross-cultural competence (mainly components k and m) also impact positively on their career adaptability development.

**Table 10:** Frequently selected components which contributed to development of career adaptability.

**Interpreter competences components**	**Mean selection rate (%)**	**Mean of importance**
i. General know-how	85.83	4.333
b. Linguistic agility in English and Chinese	83.33	4.223
a. Analytical listening and comprehension	76.67	4.140
j. Subject-specific knowledge	74.17	4.013
c. Concentration	71.67	3.743
r. Mental agility	70	3.908
e. Mastering of necessary transfer skills	66.67	3.908
u. Interpersonal relations, team work, etc.	66.67	3.526
k. Knowledge about different cultures	65	3.598
v. Meta-reflection	65	3.980
t. Professional ethics	64.17	3.708
m. Sensitivity to the communicative context	63.33	3.525
s. Preparation	63.33	3.423
d. Working memory	61.67	3.430
q. Being calm under pressure	61.67	3.558
w. Life-long learning	60	3.725

Therefore, the developmental effects of interpreter competence could be summarized as follows. In a breakdown of each adaptability resource, professionalism can substantially advance students' career concerns, and knowledge, language, and psychological competence are also conducive to this process; knowledge, language, and transfer competence are of crucial value in enhancing students' control regarding their vocational future; knowledge, cross-cultural competence, and language competence could effectively trigger students' curiosity to explore their possible selves and surroundings; and language competence constitutes the bulk of students' confidence in their future careers. To summarize, all the competence could exert favorable effects on the constructs of students' career adaptability. Among them, knowledge and language competence are most instrumental.

### Diagnosis of problems and suggestions

To address research question three, another mixed questionnaire was applied to obtain both quantitative and qualitative data to diagnose the problems and imply corresponding suggestions for the current competence-based curriculum.

Part one of the mixed questionnaire required the interpreting students to select from the same list the components that they still felt inadequate in and hence impeded the development of their career adaptability. Table 11 presents 15 frequently selected components (given the mean selection rate of the dataset, calculated as around 59.87%, only those components selected by at least 59.87% are listed). In this regard, the participants' top inadequacies were knowledge competence and language competence, as evidenced by the high selection rate of components I (93.33%), b (90%), j (80%), and a (76.67%). This might be the result of the programs' assumption that students have mastered Chinese and English upon entering and that there is no need to enhance their language. Another major inadequacy was cross-cultural competence, as seen in components k (83.33%), m (76.67%), and l (73.33%), which might be explained by the current under-representation of cross-cultural training in the current Chinese curriculum (Li, 2016). Moreover, other frequently selected components were mostly related to professionalism and psychological and strategic competence. These findings present a clue as to where more attention should be directed so as to further improve students' career adaptability.

**Table 11:** Frequently selected components participants felt still inadequate in that impeded development of career adaptability.

**Interpreter competences components**	**Mean selection rate (%)**
i. General know-how	93.33
b. Linguistic agility in English and Chinese	90
k. Knowledge about different cultures	83.33
j. Subject-specific knowledge	80
w.Life-long learning	80
a. Analytical listening and comprehension in English and Chinese	76.67
m. Sensitivity to the communicative context	76.67
l. Awareness of bridging the cross-cultural differences	73.33
v. Meta-reflection	73.33
u. Interpersonal relations, team work, etc.	70
t. Professional ethics	66.67
r. Mental agility	63.33
s. Preparation	63.33
o. Culture-specific strategies	60
q. Being calm under pressure	60

**Figure 3:**
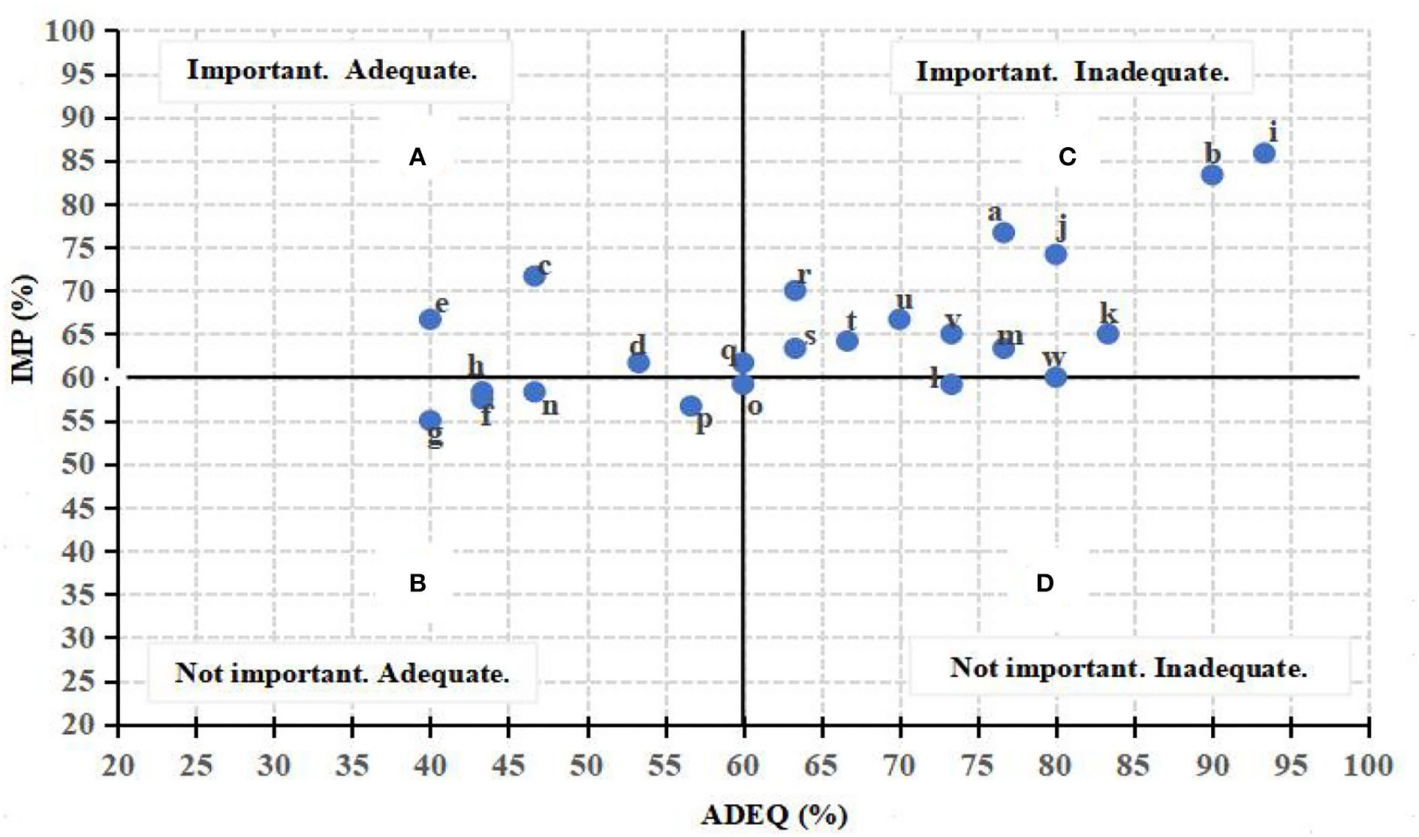
A scatterplot of the interpreter competence components.

To better identify the problems in and imply corresponding suggestions for the current competence-based curriculum, students' mean selection rates for each component that they deemed as important to facilitate and felt still inadequate to slow their career adaptability development were calculated and plotted on a scatterplot graph of importance vs. inadequacy by running SPSS 22 (Figure 3). Figure 3 demonstrates four areas: A, B, C, and D. As displayed, the components generally fell into three areas. Each area represents certain relations and implies different pedagogical suggestions.

Components in area A were considered to be important in career adaptability development by students, and they were competent in them. Three components fell into area A and all were related to transfer competence (components c, d, and e). This means that the current curriculum is already successful in training core interpreting skills. Thus, to better improve students' career adaptability, the instructor may further enhance these skills by following the current practice.

Components in area B were considered to be not, especially, important in career adaptability development by students, and they were competent in them. Five components fell into area B, mostly related to transfer competence (components g, h, and f) and strategic competence (components n and p). This again implies that the current curriculum already works well in training interpreting skills and strategies. Moreover, in view of enhancing students' career adaptability, the instructor may follow the existing practice and consider not spending additional time on these components.

Components in area C were considered to be important in career adaptability development by students, and they were not competent in them. Eleven components fell into this area. Among them, language competence (components a and b) and knowledge competence (components i and j) were the most important but with the least adequacy. Moreover, most others were related to professionalism (components s, t, u, v, and m). Another two were components of cross-cultural competence (k) and psychological competence (r). This indicates that the current curriculum is insufficient in training these competence components, and they should be given high priority in future modifications.

Components l and w were situated close to the divide between areas C and D. Component o was on the divide between areas B and D. Components in area D were not considered to be important in career adaptability development by students, and they were not competent in them. In response, the instructor should emphasize and demonstrate the importance of cross-cultural awareness and life-long learning for students in order to prevent the phenomenon wherein students may be blind to the value of these components because of their inadequacies.

As analyzed above, in the local context of China, although students have acquired sufficient transfer and strategic competence throughout the MTI programs, interpreting students still found the development of career adaptability hindered by their inadequacies in language and knowledge competence, professionalism (components s, t, u, v, and m), and cross-cultural (k) and psychological competence (r). Hence, to further develop their career adaptability, interpreting students require more language and knowledge enhancement, more cross-cultural and mental agility training, and greater professionalism.

To validate the results of the quantitative data, part two of the mixed-method questionnaire was intended to collect students' curriculum modification suggestions in view of further improving their career adaptability. Content analysis, a research method for the subjective interpretation of textual data through systematic coding and theme identification (Hsieh and Shannon, 2005; Mayring, 2021; Robinson, 2022), was applied. The coding categories and themes in this study emerged from a thematic examination of the data, rather than being determined beforehand. Table 12 presents the participants' actual response examples (factual coding), the themes (axial coding), as well as the corresponding frequencies of mention.

**Table 12:** Students' suggestions for curriculum revision.

**Actual response examples** **(Factual coding)**	**Themes (Axial coding)**	**Frequencies of mentioning**
Example 1: limited knowledge restricted my adaptation to career. Can we have a course named encyclopedia knowledge? Example 2: my main disadvantage in career was insufficient extra-linguistic knowledge, especially knowledge on certain subject and culture. The program should be modified in this direction.	Knowledge enhancement	24
Example 1: my main weaknesses were analytical listening, which substantially hampered my career adaptability. I hope the program could offer some elected courses in this regard. Example 2: my English and Chinese were not very good. So, I was not very confident. I wish I could have more language training, especially in listening and logic analysis.	Language enhancement	21
Example 1: the program should offer more simulated courses, because some skills could be only gained in contextualized way and they are important for career adaptability. Example 2: interpreting courses could offer more project tasks, which allow us to cooperate and know more about the career.	Simulated and project-based instruction	20
Example 1: we haven't had any career or entrepreneurship training so far. Specialized courses could be offered. Example 2: career adaptability is closely related to entrepreneurship mindset. Relevant learning could make us better prepared.	Entrepreneurship instruction	18
Example 1: the current internship cannot offer us much interpreting-related work. Most time I have to do administrative work. I hope the program could offer interpreting-related internship. Example 2: I failed to gain more experiences related to interpreting career in the internship. Hope we can have more opportunity to practice our professional interpreting skills in the internship.	Authentic internship	17
Example 1: the program is a little bit away from the industry. If we can have more introduction and links with the industry, our career adaptability could be enhanced. Example 2: the program should offer opportunities for us to observe the real interpreting scene, or invite those professional interpreters to give us some career mentoring.	Industry expansion	16
Example 1: although I mentioned a lot suggestions, the premise is that we should not reduce our practices of interpreting skills. They are the core of our career adaptability. Example 2: the current program runs well in training the skills and more can be done in the future.	Adequate skills training	11

In consideration of all the suggested inputs, the students expected the program to be modified in the following respects: 1. offer more language enhancement courses, 2. add more knowledge enhancement courses, 3. conduct simulated and project-based instruction, 4. enhance entrepreneurship instruction, 5. provide authentic internship opportunities, 6. expand connections with the industry, and 7. ensure adequate training of core interpreting skills. To a larger extent, the qualitative findings have verified the above quantitative results.

## Implications for curriculum modification

Based on the survey results presented above, five areas of modification may be taken into consideration so that the current competence-based curriculum can better develop interpreting students' career adaptability.

First, language enhancement courses should be added to the current curriculum. At present, no such courses are offered. This is based on the program's assumption that students have mastered Chinese and English upon entering and there is no need to enhance their language. However, the survey indicates that language competence (for example, linguistic agility and analytical listening) was most useful in developing students' control, curiosity, and confidence, and students obviously found their bilingual preparation not sufficient during the program (which might have been because English is used as a foreign language by students in the Chinese educational context). Furthermore, language competence is the starting point in the acquisition of other interpreter competence, which would again exert a lasting effect on students' interpreting careers (Yan et al., 2010; Mayor, 2015; Carrasco Flores, 2021). Thus, given these profiles, curriculum developers should consider adding language enhancement courses for the better development of students' career adaptability.

Second, knowledge enhancement modules should be available to interpreting students. As indicated above, knowledge competence (general know-how and subject-specific knowledge) is conducive to shaping all four adaptability resources, and a lack of it was seen as the greatest challenge for students' development on this front. Efforts could be made flexibly: in teaching specialized interpreting courses, we should also teach as much related background knowledge as possible, in addition to skills; aside from interpreting-related courses, we could also offer specialized knowledge modules, for instance, general knowledge lecture series, subject-specific courses, or elective knowledge courses from other programs. We also need to encourage students to read more about a diversity of topics and gradually accumulate such meta-knowledge in their daily lives (Gafiyatova and Pomortseva, 2016; Center, 2021; Gile, 2021).

Third, connections between interpreting education and industry should be established and incorporated into the curriculum. This is in consideration of the fact that competence components related to professionalism, psychology, and other non-linguistic dimensions are proven to be effective instruments to boost students' adaptability constructs (in particular, concern and confidence), and that they may be better equipped when dealing with contextualized situations (Li, 2018b; Bezzaoucha, 2021). Education–industry connections could be built at three levels. Pedagogical progression should be associated with industrial practices, for instance, project-based or simulated teaching; authentic internships, and work placements should be implemented by using external collaborators to source opportunities for real-life interpreting tasks; co-curricular industrial activities could also be organized, such as input from interpreting practitioners through workshops and seminars, exposure to a range of representatives from the professional world, or other industrial mentoring. These connecting practices could improve students' professionalism and in turn enhance their critical adaptability, as demanded by the career (Chouc and Calvo, 2010; Tang, 2020; Liu, 2022).

Fourth, cross-cultural competence deserves no less pedagogical attention than other interpreter competences. According to the survey, cross-cultural competence accounts for an overwhelming proportion in students' curiosity constructs. Furthermore, given the absence of specialized cross-cultural training in most Chinese MTI programs, its instrumental value upon adaptability development may still be uncovered. Hence, in order to make Chinese interpreting students more culturally sensitive and career-adaptable, specialized culture-related electives or modules should also be taken into consideration in curriculum development (Li, 2016; Benjamin et al., 2021; Reichertz, 2021).

Fifth, entrepreneurship factors should also be integrated into the curriculum. Entrepreneurship, the key transversal competence applicable across all spheres of life (Seikkula-Leino et al., 2021; Donoso-González et al., 2022), is believed to be beneficial for the advancement of interpreters' professionalism (Álvarez-Álvarez and Arnáiz-Uzquiza, 2017). As investigated, professionalism is of substantial instrumental value to strengthen students' concern and confidence regarding their future careers. However, as several studies show, entrepreneurial skills are not included in MTI programs (Álvarez-Álvarez and Arnáiz-Uzquiza, 2017; Galán-Mañas and Olalla-Soler, 2021), even in work placements (Olalla-Soler, 2018). Thus, it is of great necessity for curriculum developers to introduce entrepreneurship-related content throughout the program.

It is noteworthy that transfer competence also plays a substantial role in interpreting students' development of career adaptability, particularly in control and confidence. The current curriculum has worked rather well in training this core competence. As required by the student participants, embedding the above factors into the curriculum should not lead to a decrease in the content directly related to the training and practice of interpreting skills.

## Conclusion

This exploratory case study aimed at surveying interpreting students' career adaptability development, investigating the developmental effects of different interpreter competence on major adaptability resources, and using the results to inform curriculum modifications.

The survey indicates that, throughout the program, interpreting students became more concerned and well prepared for their future (concern), more curious to explore themselves and their surroundings (curiosity), and more capable of solving problems (confidence). However, their perceived beliefs and optimism toward their career construction (control) failed to develop to a satisfactory level. In addition, the study further examined the developmental effects of different interpreter competences in this process, demonstrating that all the competences could exert favorable effects on constructs of Chinese interpreting students' career adaptability, among which knowledge and language competence are most instrumental. To further boost their adaptability constructs, the results suggest that students' language and knowledge competence, professionalism, and cross-cultural and mental agility need to be further improved. As response, the current curriculum may undergo the following modifications: language enhancement courses should be added, knowledge enhancement modules should be made available, connections between interpreting education and industry should be established, cross-cultural competence should be given more pedagogical attention, and entrepreneurship factors should also be integrated.

Nevertheless, some limitations must be acknowledged for future research. First, the nature of a case study keeps the findings of the current exploration from being generalized to a wider context. Future longitudinal studies to involve more than one grade of interpreting students from diverse universities may confirm or revise the current findings. Second, the study did not consider the variables of local context and changes in time. Comparative studies based on different contexts and times may reveal the impact of local context and changes in time on career adaptability development. Third, curriculum modifications suggested in this study still lack verification. The effectiveness of the suggested curriculum modifications, with the addition of language, knowledge, and cultural-related courses and connections between industry and entrepreneurship training, would be interesting for future exploration.

The study contributes to the interpreting literature in several ways, as its findings will add to the research stream on interpreting students' career adaptability and add knowledge to the curriculum design. This study also highlights the need to investigate the intersection between career adaptability and interpreter competences, which has not been extensively explored in the current literature. As an initial effort, this study can be a foundation for further studies that could produce additional empirical evidence to bridge the gap between employability and interpreter training.

## Data availability statement

The original contributions presented in the study are included in the article/supplementary material, further inquiries can be directed to the corresponding author.

## Ethics statement

The studies involving human participants were reviewed and approved by Ethics Committee of School of Foreign Languages of Central South University, Ningxia University and Hainan University. The participants provided their written informed consent to participate in this study.

## Author contributions

ST is responsible for designing the research experiment, analyzing the dataset, and writing the thesis. ZZ and LJ are responsible for collecting the data. All authors contributed to the article and approved the submitted version.

## Funding

This research was supported by the National Social Science Foundation of China (21CYY004).

## Conflict of interest

The authors declare that the research was conducted in the absence of any commercial or financial relationships that could be construed as a potential conflict of interest.

## Publisher's note

All claims expressed in this article are solely those of the authors and do not necessarily represent those of their affiliated organizations, or those of the publisher, the editors and the reviewers. Any product that may be evaluated in this article, or claim that may be made by its manufacturer, is not guaranteed or endorsed by the publisher.
